# Asian Older Adults, Trauma, Resilience, and Health: Recent Findings From the Rutgers Asian RCMAR

**DOI:** 10.1093/geroni/igab046.192

**Published:** 2021-12-17

**Authors:** XinQi Dong, Melissa Simon, Bei Wu

## Abstract

U.S. Asians are the fastest growing group of older adults in the nation. However, there remains a dearth of disaggregated research for this population assessing health outcomes such as cognition, suicidality, mortality, and the influence of nutrition on chronic conditions. Drawing on the research of Rutgers Asian RCMAR Scientists, this symposium will examine these areas to provide a better understanding of the health of diverse groups of U.S. Asian older adults. Session 1 will assess the association between living in an ethnic enclave and better cognition among Chinese older immigrants and examine the influence of moderating factors. Session 2 will explore the prevalence of traumatic experience and discuss the association among trauma experience, lifetime mental disorder, and risk of endorsed suicide ideation among aging Asians. Session 3 will assess the relationship between family types and 6-year mortality among U.S. Chinese older adults in Chicago. Session 4 will examine the association between ultra-processed foods and cardiometabolic health (obesity, hypertension, high cholesterol, and diabetes) among U.S. adults 50 or older reporting a single ethnicity. In summation, this symposium describes key research areas such as cognition, suicidal ideation, mortality, and nutrition on the overall health of U.S. older adults. The symposium addresses both risk and protective factors that influence these health outcomes and aims to inform interventions to improve the health of U.S. Asian older adults in the areas of trauma, resilience, and health.

